# Sedentary Time and Current Tinnitus in Midlife Korean Women (Aged 40 to 64 Years): A Cross-Sectional Analysis from KNHANES 2014–2018

**DOI:** 10.3390/healthcare14183094

**Published:** 2026-09-20

**Authors:** Mikyung Ryu

**Affiliations:** Department of Alternative Medicine, Graduate School, Kyonggi University (Seoul Campus), 24 Kyonggidae-ro 9-gil, Seodaemun-gu, Seoul 03746, Republic of Korea; kyung8545kgu@gmail.com

**Keywords:** sedentary behavior, sitting time, tinnitus, women, middle aged, hearing, cross-sectional study, complex survey, KNHANES

## Abstract

**Highlights:**

**What are the main findings?**

- In midlife Korean women aged 40 to 64 years, longer daily sedentary time was associated with a higher prevalence of current tinnitus across quartiles of sitting time (highest versus lowest quartile-adjusted prevalence ratio 1.29; trend *p* < 0.001).
- The association changed little after adjustment for auditory confounders and for perceived stress, depressive symptoms, physician-diagnosed depression, sleep duration, menopausal status, and non-occupational noise exposure (adjusted prevalence ratio of 1.27 in the fully adjusted model).

**What are the implications of the main findings?**

- Sedentary behaviour may be a modifiable behavioural correlate of tinnitus, but the absolute difference is modest (about 5.6 percentage points across the exposure range) and the design is cross-sectional; the finding is hypothesis-generating and does not support clinical recommendations.
- Because tinnitus in later life is increasingly attributable to age-related hearing loss, midlife may be a more informative window in which to look for modifiable behavioural correlates.

**Abstract:**

**Background/Objectives:** Tinnitus becomes more common with age, and age-related hearing loss accounts for an increasing share of cases in later life, whereas modifiable behavioural factors may contribute relatively more in midlife. Population-based evidence linking sitting time—a construct distinct from physical inactivity—to tinnitus is limited and has not accounted for psychological and sleep-related correlates. **Methods:** We analysed Korean women aged 40–64 years in the Korea National Health and Nutrition Examination Survey (KNHANES) 2014–2018, a nationwide complex-sample survey (N = 7239; current tinnitus prevalence 21.9%). Self-reported daily sedentary time was categorised into survey-weighted quartiles (cut-points 5, 7, and 10 h/day). Survey-weighted modified-Poisson regression estimated adjusted prevalence ratios (aPRs), adjusting for demographic, socioeconomic, behavioural, aerobic-activity, and auditory factors; further models added perceived stress, depressive symptoms, physician-diagnosed depression, sleep duration, menopausal status, and non-occupational noise. **Results:** Tinnitus prevalence rose across quartiles (weighted 19.7%, 21.6%, 23.2%, and 24.9%; model-standardised 19.4%, 21.9%, 23.5%, and 24.9%). Relative to the lowest quartile, aPRs were 1.13 (95% confidence interval 0.98–1.31), 1.21 (1.07–1.36), and 1.29 (1.11–1.49; *p* = 0.0007), with a trend of 1.09 per quartile (*p* = 0.0001). The estimate was 1.26 (1.09–1.46) after mental-health and sleep adjustment, and 1.27 (1.10–1.47) when fully adjusted. A similar association was seen in men (1.22, 1.03–1.45; sedentary-by-sex interaction *p* = 0.93); in women aged 65 years and over it was absent (0.86, 0.74–1.01). **Conclusions:** Longer sedentary time was associated with a modestly higher prevalence of current tinnitus, not accounted for by the measured covariates. The design is cross-sectional, the absolute difference was small (5.6 percentage points), and reverse causation cannot be excluded; prospective confirmation is required.

## 1. Introduction

Tinnitus, the perception of sound without an external source, is common: a meta-analysis of 83 population studies estimated a pooled adult prevalence of 14.4% (95% confidence interval [CI] 12.6–16.5), with severe tinnitus in 2.3% [1]. Prevalence increases steeply with age—9.7% at 18–44 years, 13.7% at 45–64 years, and 23.6% at 65 years and over—and does not differ appreciably by sex [1]. Our own data show the same gradient in Korea (Section 3.1). In adults, the commonest identified antecedents are cochlear injury from age-related hearing loss and from occupational or recreational noise; other recognised contributors include ototoxic medication, middle-ear and other otological disease, head and neck injury, and cardiometabolic and psychological factors, although in a large proportion of cases no single cause is identifiable [2,3]. Clinically, tinnitus matters mainly when it is bothersome, and when it disturbs sleep, concentration, and emotional well-being [2,3].

Because age-related hearing loss accounts for a growing share of tinnitus after about 65 years of age, a modifiable behavioural correlate that operates in midlife may be harder to detect in older age groups, where a strong and near-universal auditory background is present [1,2,3]. Midlife may therefore be a more informative window in which to examine modifiable behavioural correlates. We restricted the analysis to women for three reasons: female-specific factors, including menopausal transition and hormonal changes, have been linked to tinnitus in Korean survey data and are best examined within one sex [4,5]; sitting time and the distribution of occupational noise exposure differ substantially between Korean men and women, so a pooled analysis would require sex-specific modelling throughout; and the resulting single-sex estimate is directly comparable with the existing Korean female-specific literature [4,5]. We report the corresponding estimate in men as an auxiliary analysis so that readers can judge whether the association is sex-specific.

Among lifestyle exposures, physical activity has been studied most often. In the U.S. National Health and Nutrition Examination Survey (NHANES), adults meeting physical-activity thresholds had a lower prevalence of tinnitus [6], and a domain-specific NHANES analysis reported lower odds of tinnitus with higher non-work physical activity and higher odds with higher work-related physical activity [7]. Clinical studies have reported lower tinnitus loudness and severity among more physically active patients [8]. Sedentary behaviour, however, is a different exposure. Defined as any waking behaviour in a sitting, reclining, or lying posture with low energy expenditure, it is epidemiologically separable from physical inactivity: a person can meet activity guidelines yet still accumulate many hours of sitting [9], and prolonged sitting is associated with adverse cardiometabolic risk markers and mortality partly independently of moderate-to-vigorous activity [9,10]. Through cardiometabolic dysregulation, low-grade inflammation, and altered autonomic and microvascular function, prolonged sitting is biologically plausible as a contributor to cochlear and auditory-pathway compromise.

Sitting time has been examined in relation to tinnitus, but the evidence base is small. In a Belgian study of 3004 participants (2751 people with tinnitus and 253 controls) using the long-form International Physical Activity Questionnaire, sitting for more than 7 h per day was associated with markedly higher odds of tinnitus (odds ratio 2.37, *p* < 0.001) [11]. That study provides important initial evidence, but its design—a comparison of a large clinical tinnitus group with a small control group—makes the magnitude of the estimate difficult to transfer to a general population, and it did not adjust for hearing status or occupational noise. A recent scoping review of dietary, anthropometric and lifestyle factors similarly concluded that the available tinnitus literature is heterogeneous, largely observational, and inconsistently adjusted for hearing loss, psychological distress, and general health status [12].

The present study was designed to address those specific gaps rather than to claim a new exposure. Its contribution is (i) a nationally representative, survey-weighted general-population estimate rather than a clinic-based one; (ii) estimation of prevalence ratios rather than odds ratios, which overstate prevalence ratios for an outcome as common as tinnitus; (iii) adjustment for the dominant auditory confounders (self-rated hearing, workplace noise, occupation) together with non-occupational noise exposure; (iv) adjustment for the psychological and sleep-related correlates that the previous literature has largely omitted; and (v) description of the exposure across the full distribution of sitting time rather than at a single threshold. We hypothesised that longer daily sedentary time would be associated with higher prevalence of current tinnitus. Because the study was not registered, all estimates are reported as exploratory and hypothesis-generating.

## 2. Materials and Methods

### 2.1. Study Design and Reporting

This was a cross-sectional analysis of pooled Korea National Health and Nutrition Examination Survey (KNHANES) data, reported in accordance with the Strengthening the Reporting of Observational Studies in Epidemiology (STROBE) statement for observational studies [13]. The study was not prospectively registered and no analysis plan was lodged with a registry before the data were examined; all estimates are therefore exploratory.

### 2.2. Data Source, Setting, and Survey Window

KNHANES is an ongoing nationwide health-examination and interview survey conducted by the Korea Disease Control and Prevention Agency (KDCA) using a stratified, multistage, clustered probability design representative of the non-institutionalised Korean population. We used the integrated interview-and-examination weight; the nutrition module was not used.

The primary analysis used the 2014–2018 window. In the 2019 survey, the otolaryngological module was administered to a subsample: among women aged 40–64 years in 2019, only 758 of 1733 (43.7%) had an answer to the tinnitus item, and the crude prevalence among respondents was 8.7%, compared with 19.6–23.2% in each year from 2014 to 2018 (Supplementary Table S1). Self-rated hearing and workplace-noise exposure were missing for the same 975 participants. A non-significant interaction test cannot demonstrate that an outcome was measured consistently, and the 2019 item could not be harmonised with the earlier cycles. We therefore restricted the primary analysis to 2014–2018 and report the 2014–2019 estimate only as a sensitivity analysis. A further advantage of this restriction is that the non-occupational noise items (earphone use in noisy environments, non-occupational noise exposure, and impulse-noise exposure) were administered throughout 2014–2018 and could be used as covariates.

### 2.3. Participants

**Figure 1:**
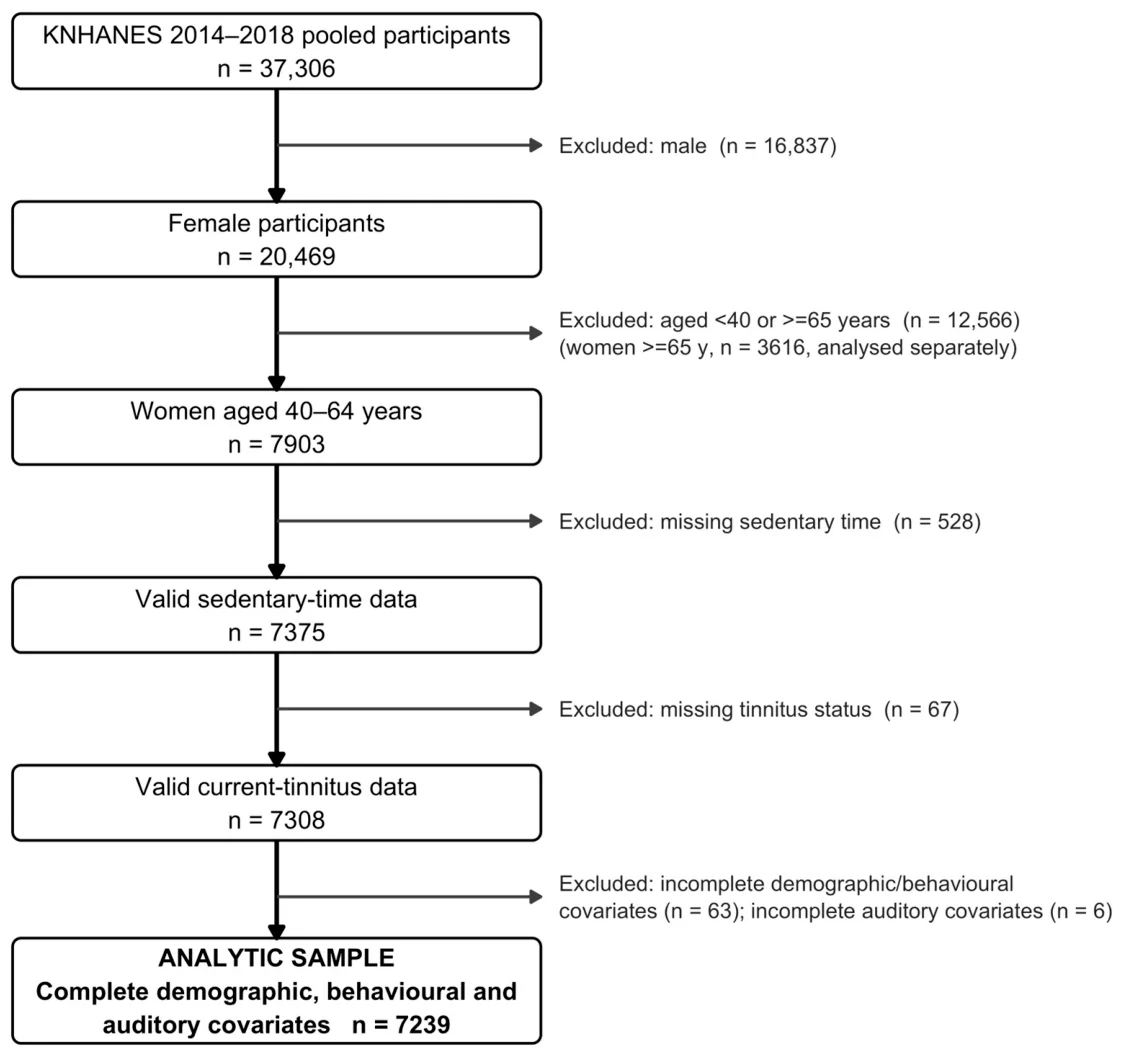
Participant flow. From 37,306 pooled Korea National Health and Nutrition Examination Survey (KNHANES) 2014–2018 participants, 20,469 were female and 7903 were aged 40–64 years; after exclusion for missing sedentary time (n = 528), missing tinnitus status (n = 67), incomplete demographic or behavioural covariates (n = 63), and incomplete auditory covariates (n = 6), 7239 women formed the analytic sample.

Eligible participants were women aged 40 to 64 years with observed sedentary time and tinnitus status and complete data on the model covariates. From 37,306 pooled participants in 2014–2018, 20,469 were female and 7903 were aged 40–64 years; 7239 had complete data for the primary model (Figure 1). Women aged 65 years or older (n = 3616) and men aged 40–64 years (n = 5306) were analysed separately as auxiliary comparisons.

### 2.4. Exposure: Sedentary Time

Daily sedentary time (hours) was derived from the physical-activity section of the health-interview questionnaire (a Global Physical Activity Questionnaire–based item) as reported hours plus minutes spent sitting or reclining on a typical day (sed = BE8_1 + BE8_2/60; “do not know/no response” codes set to missing). The item asks for total daily sitting or reclining time and does not separate occupational, transport-related, leisure-time, or screen-based sitting; domain-specific sedentary time is therefore not estimable in KNHANES, although occupation and work-related physical activity are recorded and occupation was adjusted for. Sedentary time was categorised into survey-weighted quartiles (Q1, shortest, reference, to Q4, longest; weighted cut-points 5, 7, and 10 h per day). The intervals are left-open and right-closed, so women reporting exactly 5, 7, or 10 h per day fall in Q1, Q2, and Q3 respectively; because sitting time is usually reported in whole hours, these boundary values are common (12.8%, 7.1%, and 14.2% of the analytic sample). For the trend analysis, quartiles were entered as an ordinal score (1 to 4).

### 2.5. Outcome: Current Tinnitus

Current tinnitus was defined from the ear-symptom section as a present, past-year symptom (“Have you had any noise in your ears in the past year?”; T_Q_VN = 1; codes 2 and 3 = non-case; 9 and missing = missing), a symptom-based, current definition chosen so that the outcome was time-aligned with the concurrently measured exposure.

Participants reporting tinnitus were also asked how much it interfered with daily life (three options: not bothersome; annoying/troubling; makes it difficult to sleep). We used this item to define two additional outcomes for sensitivity analysis: bothersome tinnitus (annoying or sleep-disturbing versus no tinnitus, with non-bothersome cases excluded) and sleep-disturbing tinnitus (versus no tinnitus). KNHANES does not record tinnitus duration, laterality, pitch, loudness, pulsatile versus non-pulsatile character, a validated handicap instrument such as the Tinnitus Handicap Inventory, or a clinician diagnosis of tinnitus subtype; pure-tone audiometry was last performed in KNHANES in 2010–2012, before the sedentary-time item was introduced in 2014, so audiometric thresholds and sedentary time never co-occur in the survey. Ménière disease is not ascertained in KNHANES; among chronic otological conditions, only physician-diagnosed otitis media and sinusitis were recorded, and only in 2014. Use of a hearing aid or cochlear implant was recorded throughout, and an analysis excluding users is reported.

### 2.6. Covariates

Confounders were selected using a modified disjunctive-cause criterion and a directed acyclic graph. The primary model adjusted for age (continuous), education, household-income quartile, current smoking, monthly alcohol use, aerobic physical activity (meeting the guideline), and self-rated hearing status, workplace-noise exposure, and occupation (seven categories). Self-rated hearing was treated as a confounder rather than a mediator or collider; a single self-rated item is a coarse proxy for audiometric hearing loss.

Four further covariate blocks were included, all measured in every year of 2014–2018 and essentially complete within the analytic sample (≤0.1% missing, except impulse-noise exposure at 1.5%; Supplementary Table S1):

1. Mental Health: Perceived stress (four levels: hardly any, a little, much, very much); depressive symptoms, harmonised across survey years as a Patient Health Questionnaire-9 (PHQ-9) score of 10 or higher in the years in which PHQ-9 was administered (2014, 2016, 2018) and as a report of two or more consecutive weeks of depressed mood in the intervening years (2015, 2017); and physician-diagnosed depression. A secondary analysis restricted to the PHQ-9 years additionally adjusted for the continuous PHQ-9 score. Anxiety was not measured in KNHANES 2014–2018 (the Generalized Anxiety Disorder-7 instrument was introduced later), and no insomnia or sleep-quality instrument was administered; sleep duration was the only available sleep measure.
2. Sleep: Habitual sleep duration in hours per day, harmonised across years (a single daily-average item in 2014–2015; weekday and weekend items weighted 5:2 from 2016 onwards) and categorised as <6, 6–8, and >8 h.
3. Menopausal Status: Premenopausal, postmenopausal (natural or surgical), or other/unknown.
4. Non-Occupational Noise: Earphone use in noisy environments, non-occupational noise exposure, and impulse-noise exposure.

A cardiometabolic panel (body-mass index, systolic blood pressure, fasting glucose, triglycerides, high-density-lipoprotein cholesterol, and aspartate and alanine aminotransferase) was treated as a potential mediator as well as a confounder and was therefore examined only in secondary models.

### 2.7. Complex Survey Design

All estimates incorporated the complex-sample design: the primary sampling unit as cluster, wave-unique strata defined as the interaction of survey year and stratum, and the integrated interview-and-examination weight rescaled by the number of pooled survey years (divided by 5 for 2014–2018; by 6 for the 2014–2019 sensitivity analysis; and by 3 for the PHQ-9-years analysis). Single-primary-sampling-unit strata were centred on the grand mean, and variance was estimated by Taylor linearisation. Each analytic group was defined within the pooled survey design, and the estimates were verified to be unchanged when the group was instead specified as a subpopulation (domain) of the full design, with identical adjusted prevalence ratios and identical residual degrees of freedom.

### 2.8. Statistical Analysis

Because tinnitus was common (>10%), odds ratios would overstate the prevalence ratio; we therefore estimated adjusted prevalence ratios (aPRs, the ratio of prevalence in an exposure group to that in the reference group after covariate adjustment) using survey-weighted modified-Poisson regression (log link, quasi-Poisson family) with design-based robust standard errors [14]. The primary estimand was the aPR for the longest versus shortest sedentary quartile (Q4 versus Q1), accompanied by the Q2-versus-Q1 and Q3-versus-Q1 contrasts and a per-quartile linear-trend test. Prevalence by quartile was summarised both as weighted observed prevalence and as model-standardised (predictive-margin) prevalence. Descriptive statistics used weighted means and proportions with Rao–Scott tests.

Robustness was assessed with the covariate blocks described above, with the alternative outcome definitions, with restriction to participants without diagnosed cardiovascular disease, including 2019, and in men aged 40–64 years and women aged 65 years and over (with formal interaction tests). E-values—the minimum strength of association, on the risk-ratio scale, that an unmeasured confounder would need with both exposure and outcome to explain away an observed association—were computed for the point estimate and for the confidence limit nearest the null [15]. Because all analyses are exploratory and no confirmatory hypothesis was registered, we applied no formal multiplicity correction.

Analyses were performed in R 4.6.0 with the survey and srvyr packages; a fixed seed was set, and the core estimates were independently reproduced from raw data using a separate code path. Full session information and input and output checksums are archived with the analysis.

### 2.9. Ethical Considerations

KNHANES is publicly available, de-identified secondary data released by the KDCA. The survey was approved by the KDCA Institutional Review Board (approval numbers 2013-12EXP-03-5C [2014] and 2018-01-03-P-A [2018]; the 2015–2017 surveys were conducted without separate IRB review under the Bioethics and Safety Act as national public-welfare research). All participants provided written informed consent. This secondary analysis of open, de-identified data was exempt from additional review.

## 3. Results

### 3.1. Tinnitus Prevalence by Age

**Figure 2:**
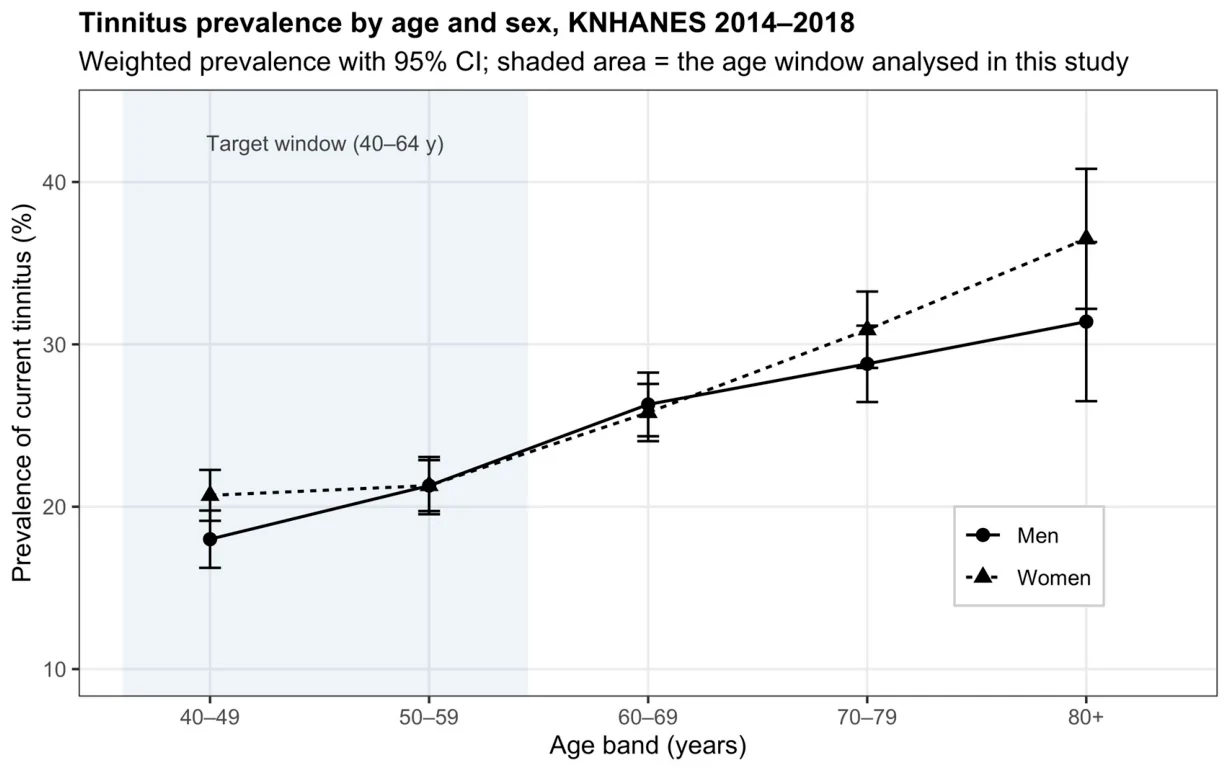
Tinnitus prevalence by age and sex, KNHANES 2014–2018. Weighted prevalence of current tinnitus with 95% confidence intervals by 10-year age band in men and women aged 40 years and over. The shaded area marks the 40–64-year window analysed in this study.

In KNHANES 2014–2018, the weighted prevalence of current tinnitus rose monotonically with age in both sexes: from 20.7% (women) and 18.0% (men) at 40–49 years to 36.5% and 31.4% at 80 years and over (Figure 2). Prevalence in the target age band (40–64 years) was 21.9% in women.

### 3.2. Participants and Descriptive Characteristics

Weighted descriptive characteristics of the analytic sample (N = 7239) are shown in Table 1. The weighted mean age was 51.4 years and mean sedentary time 7.19 h/day. Current tinnitus prevalence rose across sedentary quartiles (19.7%, 21.6%, 23.2%, and 24.9%; *p* = 0.006). Women in higher sedentary quartiles were less likely to meet the aerobic-activity guideline (51.9% to 38.2% from Q1 to Q4; *p* < 0.001), were more likely to have attained a college education or higher, and differed by occupational distribution (*p* < 0.001). Self-rated hearing did not differ across quartiles (*p* = 0.51), and workplace-noise exposure was highest in the shortest-sitting quartile (16.0% in Q1 versus 12.5% in Q4; *p* < 0.001). Higher sedentary quartiles also had somewhat more perceived stress (much or very much: 23.4% in Q1 versus 29.9% in Q4; *p* = 0.041), more depressive symptoms (8.0% versus 11.2%; *p* = 0.039), and shorter sleep (*p* = 0.002), which is why these variables were treated as candidate confounders.

### 3.3. Sedentary Time and Current Tinnitus: Primary Analysis

**Figure 3:**
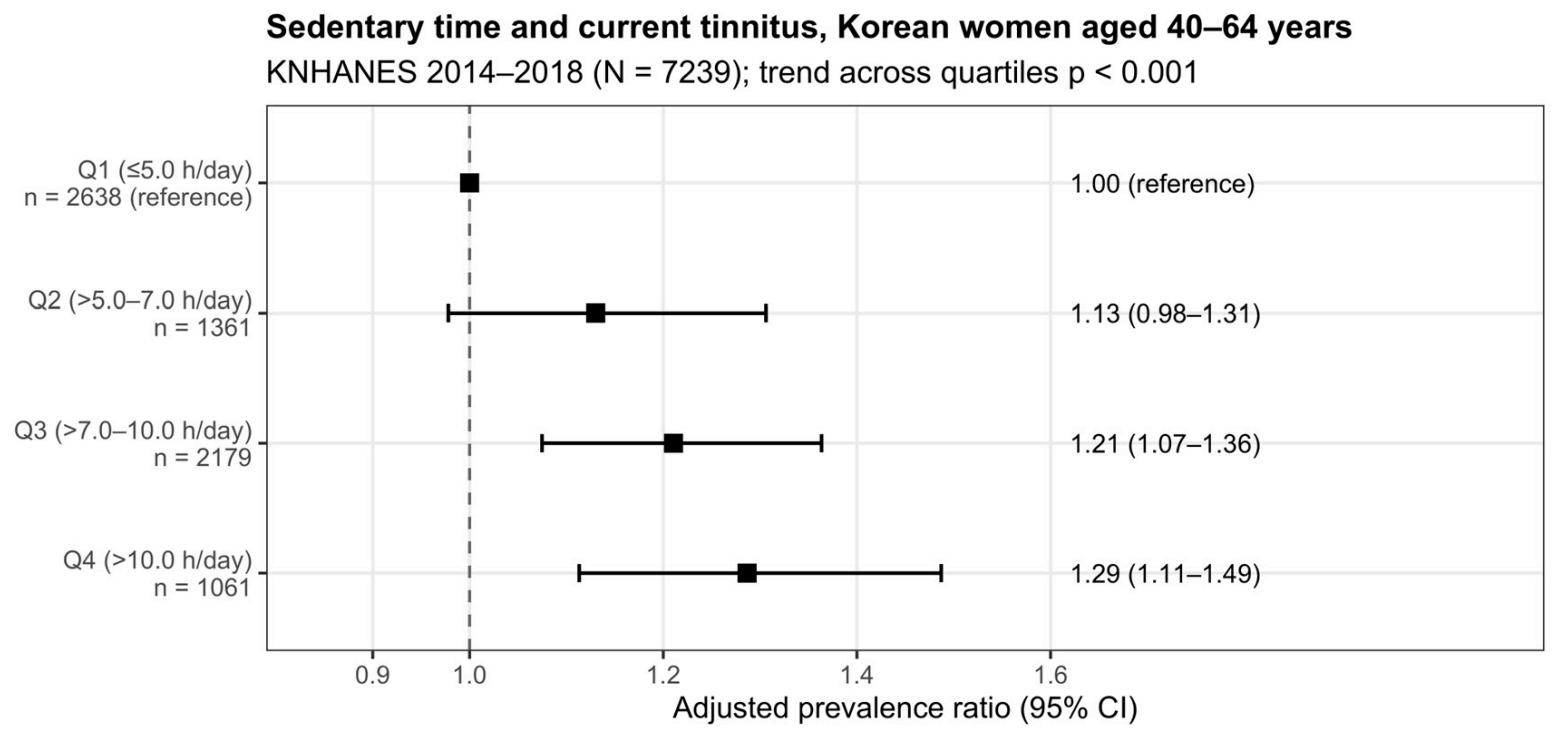
Sedentary time and current tinnitus. Adjusted prevalence ratios for current tinnitus by sedentary-time quartile with the number of participants per quartile (N = 7239). The dashed vertical line marks the null value (adjusted prevalence ratio = 1.00).

**Figure 4:**
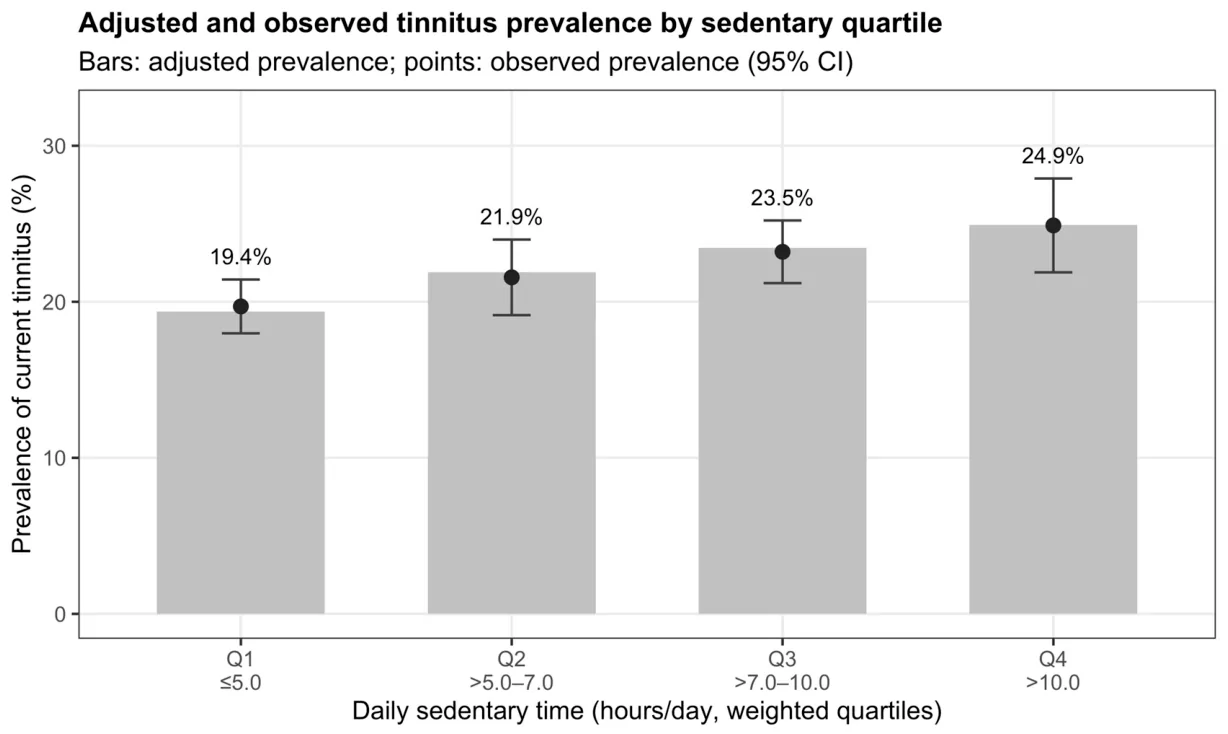
Observed and adjusted prevalence by sedentary quartile. Bars show model-standardised prevalence (19.4%, 21.9%, 23.5%, 24.9%); points with error bars show weighted observed prevalence with 95% confidence intervals.

In the primary model—adjusting for age, education, household income, smoking, alcohol use, aerobic activity, self-rated hearing, workplace noise, and occupation—longer sedentary time was associated with a higher prevalence of current tinnitus, and the association was graded across quartiles (Table 2; Figure 3). Relative to the shortest quartile (Q1), the aPR was 1.13 (95% CI 0.98–1.31, *p* = 0.097) for Q2, 1.21 (95% CI 1.07–1.36, *p* = 0.002) for Q3, and 1.29 (95% CI 1.11–1.49, *p* = 0.0007) for Q4. The per-quartile linear trend was 1.09 (95% CI 1.04–1.14, *p* = 0.0001). On the absolute scale, model-standardised prevalence rose from 19.4% in Q1 to 24.9% in Q4, a difference of 5.6 percentage points. Figure 4 displays these model-standardised prevalences together with the weighted observed prevalence and its 95% confidence interval for each sedentary-time quartile.

### 3.4. Adjustment for Mental Health, Sleep, Menopausal Status, and Non-Occupational Noise

The mental-health variables behaved as expected for tinnitus correlates in this sample: relative to women reporting hardly any stress, the aPR for tinnitus was 1.78 (95% CI 1.48–2.13) for “much” stress and 1.68 (95% CI 1.32–2.13) for “very much”; depressive symptoms carried an aPR of 1.33 (95% CI 1.16–1.53) and physician-diagnosed depression 1.43 (95% CI 1.22–1.67) (Supplementary Table S3). They were also associated with the exposure: mean sedentary time rose from 7.07 h/day in women with hardly any stress to 7.66 h/day in those reporting “very much” stress, and was 7.15 versus 7.56 h/day in women without and with depressive symptoms (Supplementary Table S4). These variables therefore satisfy the criteria for potential confounders.

Table 3 sets out the sequential-adjustment ladder and the covariate-block sensitivity analyses. Adjusting for these variables nonetheless left the exposure estimate essentially unchanged. Adding perceived stress gave an aPR of 1.26 (95% CI 1.09–1.46); depressive symptoms 1.27 (95% CI 1.10–1.46); physician-diagnosed depression 1.28 (95% CI 1.11–1.48); sleep duration 1.28 (95% CI 1.11–1.49); and all mental-health and sleep variables together 1.26 (95% CI 1.09–1.46), with a per-quartile trend of 1.08 (95% CI 1.04–1.13, *p* = 0.0004). In the years in which PHQ-9 was administered (2014, 2016, and 2018; n = 4355), adjustment for the continuous PHQ-9 score moved the estimate from 1.37 (95% CI 1.14–1.65) to 1.32 (95% CI 1.09–1.59). Adding menopausal status gave 1.29 (95% CI 1.12–1.49) and adding the three non-occupational noise items (earphone use in noisy environments, non-occupational noise exposure, and impulse-noise exposure) gave 1.29 (95% CI 1.12–1.49). The model containing all covariate blocks simultaneously gave 1.27 (95% CI 1.10–1.47).

For the primary Q4-versus-Q1 estimate, the E-value was 1.89 for the point estimate and 1.47 for the confidence limit nearest the null. An unmeasured confounder would need to be associated with both prolonged sitting and tinnitus by a risk ratio of at least about 1.47 to 1.89, over and above the measured covariates, to explain away the association. We emphasise that the E-value characterises only the strength that an unmeasured confounder would require; it is not evidence that no such confounder exists, and it does not address bias from reverse causation or measurement error.

### 3.5. Robustness: Outcome Definition, Survey Window, and Comorbidity

Among the 1573 women reporting current tinnitus, 1000 (63.6%) described it as not bothersome, 501 (31.9%) as annoying, and 72 (4.6%) as severe enough to interfere with sleep (Supplementary Table S6). Restricting the outcome to bothersome tinnitus gave an aPR of 1.23 (95% CI 0.94–1.61) and restriction to sleep-disturbing tinnitus gave an aPR of 1.33 (95% CI 0.61–2.89); both estimates were compatible with the primary estimate but were imprecise, particularly the latter, which rested on 72 cases. Excluding hearing-aid or cochlear-implant users gave 1.29 (95% CI 1.11–1.49). Including 2019 gave 1.25 (95% CI 1.08–1.44; N = 7974), and excluding women with diagnosed cardiovascular disease gave 1.29 (95% CI 1.11–1.49). All robustness analyses are listed in Supplementary Table S2 and displayed in Supplementary Figure S1.

### 3.6. Auxiliary Comparisons by Sex and Age

In men aged 40 to 64 years (n = 5306) the same model gave an aPR of 1.22 (95% CI 1.03–1.45, *p* = 0.021), and the sedentary-by-sex interaction was far from significant (*p* = 0.93); the association therefore does not appear to be specific to women, and the restriction to women should be read as a scope decision rather than as evidence of a sex-specific effect. In women aged 65 years and over (n = 3616) the association was absent (aPR 0.86, 95% CI 0.74–1.01, *p* = 0.065), and the sedentary-by-age-group interaction was significant (*p* = 0.0008). We do not interpret the older-age estimate as evidence of a protective effect of sitting; it is compatible with the null, and with several non-causal explanations discussed below.

## 4. Discussion

### 4.1. Principal Findings

In a nationally representative sample of Korean women aged 40 to 64 years, longer daily sedentary time was associated with a modestly higher prevalence of current tinnitus, graded across quartiles of sitting time (Q4 versus Q1 aPR 1.29; per-quartile trend *p* = 0.0001). The association was not materially altered by adjustment for auditory factors, cardiometabolic markers, perceived stress, depressive symptoms, physician-diagnosed depression, sleep duration, menopausal status, or non-occupational noise exposure (aPR 1.27 with all blocks included). A similar estimate was obtained in men, whereas in women aged 65 years and over the association was absent.

### 4.2. Comparison with the Prior Literature

Our findings are consistent in direction with, but considerably smaller in magnitude than, the one previous study to treat sitting time as an exposure for tinnitus. In a Belgian sample, sitting for more than 7 h per day carried an odds ratio of 2.37 [11]. That study compared 2751 people with tinnitus, largely recruited through clinical channels, with 253 controls, and did not adjust for hearing status or occupational noise; both the sampling frame and the use of an odds ratio for a very common outcome would be expected to yield a larger estimate than a survey-weighted prevalence ratio from a general population. Our estimate—aPR 1.29 for ≥10 versus <5 h per day—should be read as the general-population counterpart of that clinic-based observation, and, taken together, the two studies suggest that the association is real but more modest than the earlier figure implies. This also constitutes a European–Asian comparison, and the concordance in direction across two very different populations and questionnaires is reassuring, although we are not aware of any European general-population survey that has examined sitting time and tinnitus with survey weights.

The physical-activity literature is broadly concordant. In NHANES, higher physical activity was associated with lower tinnitus prevalence [6], and a domain-specific NHANES analysis found lower odds with non-work activity and higher odds with work-related activity, while adjusting for sedentary time as a covariate [7]; clinical work has linked greater activity to lower tinnitus loudness and severity [8]. A recent scoping review concluded that this literature remains heterogeneous and inconsistently adjusted for hearing loss and psychological distress [12]—precisely the gap the present analysis was designed to narrow.

Sedentary behaviour has also been examined in relation to other otological conditions, and that literature is instructive because it has generally been framed in the opposite causal direction. In the Chilean National Health Survey (n = 3512 adults aged ≥40 years), self-reported hearing loss was associated with high sedentary behaviour (≥8 h/day; odds ratio 1.81, 95% CI 1.16–2.82) but not with physical activity in any domain [16]; and in NHANES 2003–2006, older adults with normal audiometric hearing accumulated less accelerometer-measured sedentary time than those with moderate or greater hearing loss [17]. Both studies treated the auditory condition as the exposure and sitting as the outcome. Because the design of all these studies, including ours, is cross-sectional, the same data are compatible with either direction, and the coexistence of these two branches of literature is itself an argument for reading our estimate as bidirectional rather than as a one-way effect (Section 4.5). Our midlife prevalence (21.9%) is at the upper end of, but within, the range reported internationally [1] and is consistent with previous Korean survey estimates [4,5]. Prior Korean studies have emphasised female-specific correlates of tinnitus such as menstrual irregularity, body-mass index, and mental health [4,5]; the present analysis adds sitting time, measured and adjusted alongside those factors.

### 4.3. Interpretation and Mechanism

Several mechanisms could plausibly link prolonged sitting to tinnitus, although any mechanistic reading is speculative in a cross-sectional design. Prolonged sedentary time promotes cardiometabolic dysregulation, low-grade systemic inflammation, and altered autonomic and microvascular function [9,10], all of which could, in principle, compromise cochlear microcirculation and central auditory processing. That the estimate changed little after adjustment for blood pressure, glucose, and lipid markers suggests that, if such a pathway operates, it is not captured by these standard markers.

The contrast with older women invites caution rather than mechanistic conclusions. In later life, an increasing share of tinnitus is attributable to age-related hearing loss and other degenerative auditory changes [1,2,3], and against that strong background the relative contribution of a behavioural exposure may be diluted, so that a modest association becomes harder to detect. Rather than describing this as presbycusis “masking” the signal—wording that would overstate what a cross-sectional comparison can show—we state only that presbycusis may partly attenuate or obscure the association. Several alternative explanations are at least as plausible and cannot be distinguished with these data: residual confounding that differs by age, competing risks and differential survival, selection effects related to institutionalisation or disability, changes in the meaning of self-reported sitting time in retirement, and simple model instability. Importantly, in the present 2014–2018 analysis, the older-age estimate (0.86, 95% CI 0.74–1.01) is compatible with the null, so the age contrast rests on the interaction test rather than on a demonstrated inverse association.

The similarity of the estimate in men (1.22) and the null sedentary-by-sex interaction indicate that the association is not specific to women. The restriction of the primary analysis to women therefore defines the population to which our headline estimate applies; it is not evidence that sitting matters only in women, and the finding in men strengthens rather than weakens the overall observation by providing an internal replication.

### 4.4. Statistical Versus Clinical Significance

The association is statistically robust but clinically modest, and these two properties should not be conflated. Across the entire observed exposure range—from under 5 to at least 10 h of sitting per day, a difference of roughly 9 h—model-standardised tinnitus prevalence differed by 5.6 percentage points (19.4% versus 24.9%), which corresponds to about 18 additional people reporting tinnitus per 1000 for each additional quartile of sitting. Most of that additional tinnitus would, on the observed distribution of bothersomeness, be non-bothersome: only about one third of cases in this sample were described as annoying and fewer than 5% as sleep-disturbing, and the estimates for bothersome and sleep-disturbing tinnitus, while directionally similar, did not reach statistical significance. A difference of this size is meaningful at the population level, where sitting time is nearly universal and increasing, but it is not a basis for individual clinical counselling; no clinician should currently advise a patient that reducing sitting time will prevent or relieve tinnitus. Whether reducing sedentary time changes tinnitus occurrence or severity is a question for prospective and interventional research, not one this study can answer.

### 4.5. Reverse Causation

Reverse causation is a serious and plausible alternative explanation for our finding, and the cross-sectional design cannot exclude it. Bothersome tinnitus is associated with anxiety, depressive symptoms, sleep disturbance, impaired concentration, and a higher overall symptom burden, any of which could reduce activity and increase time spent sitting. The association we observe may therefore be bidirectional: sitting may contribute to tinnitus, and tinnitus-related distress may promote sitting. That the same association has been reported in the opposite direction for hearing loss—where sedentary time is treated as the outcome of the auditory condition [16,17]—reinforces this concern. Three observations bear on this without resolving it. First, adjustment for perceived stress, depressive symptoms, physician-diagnosed depression, and sleep duration barely moved the estimate, which makes an explanation running entirely through measured psychological distress less likely—though these are crude measures and residual mediation through distress remains possible. Second, if the pathway ran mainly from bothersome tinnitus to inactivity, one would expect the association to be strongest for bothersome and sleep-disturbing tinnitus; instead, those estimates were similar in size to the estimate for any tinnitus, and both were imprecise. Third, an association of similar size was present in men. None of these is decisive. Accordingly, we describe the association throughout as an association and not as an independent or causal effect, and we regard prospective data as necessary.

### 4.6. Strengths and Limitations

The study has several strengths. It draws on a large, nationally representative sample analysed with correct complex-survey handling, using wave-unique strata, correct treatment of the analytic subpopulation, and Taylor-linearised variance. The exposure definition distinguishes sedentary time from physical activity, and the models adjust for the principal auditory confounders together with non-occupational noise, psychological, sleep-related, and menopausal factors. We used an appropriate prevalence-ratio estimand, and we independently reproduced the core estimates through a separate code path.

The limitations, however, are substantial and constrain the interpretation.

The study is cross-sectional and was not registered, and no analysis plan was lodged before the data were examined. Temporality cannot be established and reverse causation cannot be excluded (Section 4.5). All estimates reported here are exploratory.

Measurement is a further concern, beginning with the exposure, which was self-reported through a single question on typical daily sitting or reclining time. Items of this kind correlate only moderately with accelerometer-measured sedentary time and, in most validation studies, substantially underestimate it, with errors that grow at higher levels of true sitting. If such misclassification is non-differential with respect to tinnitus, which is the more likely situation for a behaviour reported before any question about ear symptoms, the expected consequence is attenuation, so the true association may be somewhat stronger than the one we estimated. Categorisation into quartiles could nonetheless blur the contrast between adjacent categories, and differential reporting cannot be excluded if, for example, women with bothersome tinnitus recall their daily routine differently. A single item also cannot separate occupational, transport, leisure, and screen-based sitting, whose health implications may differ. We adjusted for occupation, and sedentary time did vary markedly across occupational groups, from 5.7 h/day in agricultural workers to 9.2 h/day in clerical workers (Supplementary Table S5), but domain-specific sitting is not recoverable from KNHANES, and device-based measurement in future studies would materially strengthen the evidence.

Turning to the outcome, tinnitus was likewise ascertained from a single self-reported item. We were able to characterise bothersomeness, and did so, but KNHANES records nothing on duration, laterality, pitch, loudness, pulsatile versus non-pulsatile character, tinnitus handicap, or clinical subtype, and no audiometric thresholds were measured in these survey years, so tinnitus accompanied by hearing loss cannot be separated from tinnitus without it. Ménière disease is not ascertained, and chronic otological disease is recorded only partially and only in 2014, so participants with these conditions could not be excluded. We therefore cannot determine which clinical tinnitus phenotypes the association applies to, and the possibility that it is confined to, or absent from, particular phenotypes such as bothersome tinnitus, tinnitus with hearing loss, or somatic or pulsatile tinnitus remains open; the imprecision of the bothersome and sleep-disturbing estimates directly reflects this limitation.

What could not be measured also limits the control of confounding, and residual confounding remains possible despite the added covariate blocks. Self-rated hearing is only a coarse proxy for audiometric loss, anxiety was not measured, and sleep was captured as duration alone, with no measure of insomnia or sleep quality. Medication use, including ototoxic drugs, antidepressants, and hypnotics, was not adjusted for, and menopausal status was recorded without hormone therapy being modelled. The E-value quantifies the strength an unmeasured confounder would need but does not establish that none is present.

The analytic sample and the survey coverage bound the generalisability of the findings. Analyses used complete cases without multiple imputation, and 664 of 7903 eligible women (8.4%) were excluded for missing data, most of them (528) because of a missing sedentary-time response and only 136 (1.7%) because of missing outcome or covariate data. The results apply to Korean women aged 40 to 64 years, and the auxiliary estimates in men and in older women are exploratory. Finally, the 2019 survey cycle was excluded from the primary analysis because of an apparent change in the administration of the tinnitus item, although including this did not change the conclusions.

## 5. Conclusions

In a nationally representative sample of Korean women aged 40 to 64 years, longer daily sedentary time was associated with a modestly higher prevalence of current tinnitus, graded across quartiles of sitting time, and this association was not accounted for by measured demographic, behavioural, auditory, cardiometabolic, psychological, sleep-related, or menopausal factors. The absolute difference across the exposure range was about 5.6 percentage points, and a similar association was observed in men. The design is cross-sectional and the findings are exploratory: temporality cannot be established, reverse causation remains plausible, and the results do not support advising patients that reducing sitting time will prevent or relieve tinnitus. Prospective studies with device-measured sedentary time and clinically characterised tinnitus are needed.

## Figures and Tables

**Table 1:** Weighted characteristics of Korean women aged 40 to 64 years by sedentary-time quartile, KNHANES 2014–2018 (N = 7239).

Characteristic	Q1 (≤5.0 h/d)	Q2 (>5.0–7.0 h/d)	Q3 (>7.0–10.0 h/d)	Q4 (>10.0 h/d)	Overall	*p*
N (unweighted)	2638	1361	2179	1061	7239	
Age, years, mean (SE)	51.92 (0.16)	51.93 (0.21)	50.84 (0.17)	50.86 (0.23)	51.44 (0.10)	<0.001
Sedentary time, h/d, mean (SE)	3.82 (0.03)	6.37 (0.02)	9.09 (0.02)	12.82 (0.05)	7.19 (0.05)	<0.001
Sleep duration, h/d, mean (SE)	6.95 (0.03)	6.90 (0.04)	6.94 (0.03)	6.74 (0.05)	6.91 (0.02)	<0.001
**Education, %**						<0.001
≤Elementary	16.8	13.8	11.7	12.2	14.0	
Middle school	16.9	16.0	10.6	10.6	13.9	
High school	43.7	40.8	40.4	41.6	41.9	
College or higher	22.5	29.5	37.4	35.6	30.2	
**Household income quartile, %**						<0.001
Q1 (lowest)	10.8	11.4	9.8	10.9	10.6	
Q2	27.7	24.4	20.2	21.5	23.9	
Q3	31.4	29.5	29.2	30.6	30.2	
Q4 (highest)	30.1	34.7	40.8	37.0	35.2	
Current smoker, %	4.8	4.2	4.3	7.9	5.0	<0.001
Monthly alcohol drinker, %	45.4	44.0	42.5	43.3	44.0	0.336
Meets aerobic PA guideline, %	51.9	46.2	42.6	38.2	46.1	<0.001
**Self-rated hearing, %**						0.509
Good	88.8	90.8	89.4	90.0	89.5	
Fair	9.6	8.0	8.9	8.9	9.0	
Poor	1.4	1.1	1.6	1.1	1.4	
Very poor	0.2	0.1	0.0	0.0	0.1	
Workplace-noise exposure, % yes	16.0	11.0	10.1	12.5	12.8	<0.001
Earphone use in noisy places, % yes	7.7	7.1	8.1	10.1	8.1	0.085
Non-occupational noise exposure, % yes	2.3	1.9	1.3	1.3	1.8	0.110
Impulse-noise exposure, % yes	9.2	8.7	7.8	8.1	8.5	0.484
**Occupation (7-category), %**						<0.001
Managers and professionals	8.1	12.3	15.8	11.5	11.7	
Clerical/office workers	3.6	4.4	13.1	16.0	8.4	
Service and sales workers	27.7	23.1	15.6	14.1	21.2	
Skilled agriculture/forestry/fishery	3.7	3.0	1.5	0.9	2.5	
Craft and machine-operation workers	4.4	2.2	4.7	6.5	4.4	
Elementary/manual labourers	15.7	12.9	8.2	7.2	11.7	
Not economically active	36.7	42.2	41.1	43.8	40.1	
**Perceived stress, %**						0.041
Hardly any	14.3	14.3	13.1	13.0	13.7	
A little	62.4	62.4	60.5	57.1	61.0	
Much	19.0	18.8	21.2	23.3	20.2	
Very much	4.4	4.4	5.2	6.6	5.0	
Depressive symptoms, %	8.0	7.7	8.9	11.2	8.7	0.039
Physician-diagnosed depression, %	6.6	6.0	6.0	7.6	6.5	0.411
**Sleep duration, %**						0.002
<6 h	14.7	15.9	13.5	17.5	15.0	
6–8 h	58.7	60.0	61.7	62.8	60.5	
>8 h	26.6	24.0	24.8	19.6	24.6	
**Menopausal status, %**						<0.001
Premenopausal	43.5	41.6	48.4	49.2	45.5	
Postmenopausal	55.0	57.3	49.8	48.3	52.9	
Other/unknown	1.5	1.1	1.7	2.5	1.6	
Current tinnitus, %	19.7	21.6	23.2	24.9	21.9	0.006

Values are weighted mean (SE) for continuous variables or weighted % for categorical variables; N is unweighted. *p* from design-based tests (Wald test for continuous variables; Rao–Scott second-order-corrected χ^2^ for categorical variables), complex-survey weighted (weight = wt_itvex/5, strata = interaction[year, kstrata], PSU = psu). PA, physical activity; PSU, primary sampling unit; SE, standard error. Sedentary-time quartile cut-points are 5.0, 7.0, and 10.0 h/day. Bold row labels denote categorical variable headings; the rows beneath each give its categories.

**Table 2:** Adjusted prevalence ratios for current tinnitus by sedentary-time quartile for Korean women aged 40 to 64 years, KNHANES 2014–2018 (N = 7239).

Comparison	n	Weighted Prevalence, %	Model-Standardised Prevalence, %	aPR	95% CI	*p*	E-Value, Point (CI Limit)
Q1 (≤5.0 h/d)	2638	19.7	19.4	1.00 (ref.)	—	—	
Q2 (>5.0–7.0 h/d)	1361	21.6	21.9	1.13	0.98–1.31	0.097	
Q3 (>7.0–10.0 h/d)	2179	23.2	23.5	1.21	1.07–1.36	0.002	
Q4 (>10.0 h/d)	1061	24.9	24.9	1.29	1.11–1.49	0.0007	1.89 (1.47)
Linear trend, per quartile †				1.09	1.04–1.14	0.0001	

aPR, adjusted prevalence ratio (survey-weighted modified-Poisson regression, design-based robust standard errors); CI, confidence interval. Model adjusted for age, education, household-income quartile, current smoking, monthly alcohol use, aerobic physical activity, self-rated hearing, workplace-noise exposure, and occupation. E-values are the minimum risk ratio an unmeasured confounder would need with both exposure and outcome to explain away the association, as reported for the Q4-versus-Q1 estimate. † aPR per one-quartile increment in an ordinal sedentary score.

**Table 3:** Sequential adjustment and covariate-block sensitivity analyses: Q4-versus-Q1 adjusted prevalence ratio for current tinnitus in Korean women aged 40 to 64 years, KNHANES 2014–2018.

Model	aPR (Q4 vs. Q1)	95% CI	*p*
M0 Crude	1.26	1.09–1.46	0.001
M1 + age, education, income, smoking, alcohol, aerobic activity	1.31	1.13–1.51	0.0003
M2 + self-rated hearing, workplace noise, occupation (**primary model**)	1.29	1.11–1.49	0.0007
M3 + cardiometabolic panel	1.30	1.12–1.50	0.0004
M4 + perceived stress, depressive symptoms, physician-diagnosed depression, sleep duration	1.26	1.09–1.46	0.002
M5 + menopausal status	1.29	1.12–1.49	0.0006
M6 + earphone use, non-occupational noise, impulse noise	1.29	1.12–1.49	0.0006
M7 + all of the above simultaneously	1.27	1.10–1.47	0.001

Models M0 to M3 are cumulative. Models M4 to M6 each add the listed covariates to M2, the primary model, and M7 includes all covariate blocks simultaneously. The analytic sample is N = 7239 for M0 to M2 and for M5; it is smaller where a covariate block is incomplete (M3, n = 6998; M4, n = 7228; M6, n = 7122; M7, n = 7111), as listed in Supplementary Table S2. aPR, adjusted prevalence ratio; CI, confidence interval. Bold denotes the pre-specified primary model (M2).

## Data Availability

KNHANES data are publicly available from the Korea Disease Control and Prevention Agency (https://knhanes.kdca.go.kr, accessed on 6 July 2026). Analysis code is available from the corresponding author on reasonable request.

## Supplementary Materials

The following supporting information can be downloaded at: https://www.mdpi.com/article/10.3390/healthcare14183094/s1, Figure S1: Forest plot of all adjusted prevalence ratios; Table S1: Availability and missingness of study variables by survey year, Korean women aged 40 to 64 years, KNHANES 2014–2019; Table S2: Complete robustness analyses (Q4 versus Q1 adjusted prevalence ratios); Table S3: Mental-health, sleep, and menopausal variables as correlates of current tinnitus in the primary model; Table S4: Mean daily sedentary time by mental-health, sleep, and menopausal status; Table S5: Sedentary time and tinnitus prevalence by occupational category; Table S6: Distribution of tinnitus bothersomeness among women reporting current tinnitus.

## Abbreviations

The following abbreviations are used in this manuscript:

aPR	adjusted prevalence ratio
CI	confidence interval
KDCA	Korea Disease Control and Prevention Agency
KNHANES	Korea National Health and Nutrition Examination Survey
NHANES	National Health and Nutrition Examination Survey (United States)
PA	physical activity
PHQ-9	Patient Health Questionnaire-9
STROBE	Strengthening the Reporting of Observational Studies in Epidemiology
