# Supplementary material for: Sedentary Time and Current Tinnitus in Midlife Korean Women (Aged 40 to 64 Years): A Cross-Sectional Analysis from KNHANES 2014–2018
Source: Healthcare (Basel). 2026 Sep 20;14(18):3094. doi: 10.3390/healthcare14183094 (PMC13606051; doi:10.3390/healthcare14183094)
Supplement: Supplementary file 1 [file healthcare-14-03094-s001.zip › healthcare-4454308-supplementary.pdf]

Supplementary Materials

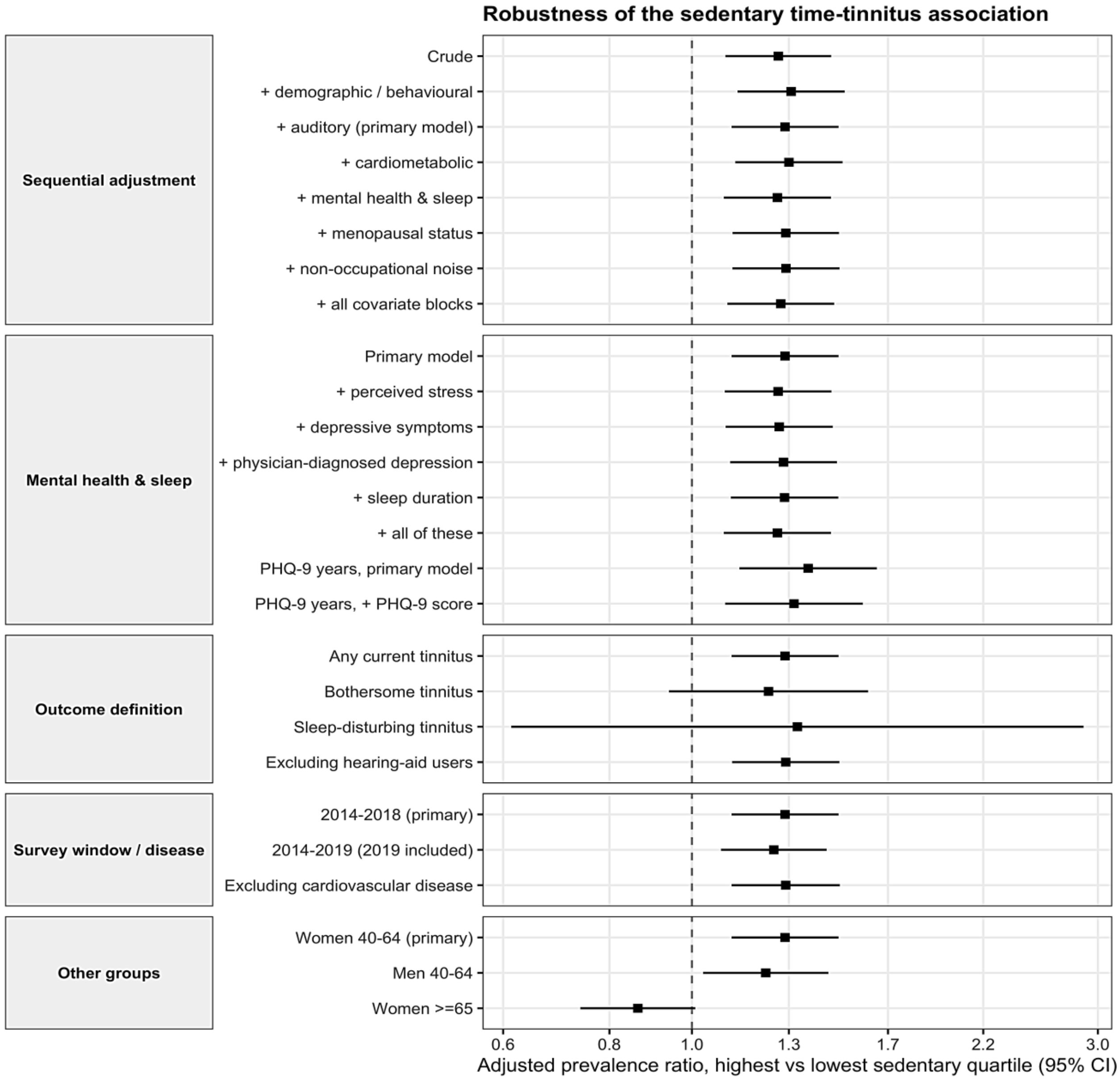

Figure S1. Forest plot of all adjusted prevalence ratios listed in Table S2, grouped by sequential adjustment, mental-health and sleep adjustment, outcome definition, survey window and comorbidity, and auxiliary groups.

Table S1. Availability and missingness of study variables by survey year, Korean women aged 40 to 64 years, KNHANES 2014–2019

Survey year	Women aged 40–64 y (n)	Tinnitus item answered, n (%)	Crude tinnitus prevalence, %	Self-rated hearing answered, n	Perceived stress missing, %	Depressive-symptom instrument	Depressive symptoms missing, %	Physician-diagnosed depression missing, %	Sleep duration missing, %	Non-occupational noise block missing, %
2014	1541	1442 (93.6)	21.0	1442	9.1	PHQ-9 (≥10)	13.5	12.8	9.1	7.3
2015	1589	1506 (94.8)	23.2	1506	6.4	2-week depressed mood	6.4	11.6	6.5	6.8
2016	1627	1558 (95.8)	23.2	1558	4.3	PHQ-9 (≥10)	7.2	6.8	7.1	4.5
2017	1693	1607 (94.9)	20.6	1607	4.6	2-week depressed mood	4.5	8.2	8.5	8.8
2018	1720	1657 (96.3)	19.6	1657	3.2	PHQ-9 (≥10)	6.3	5.9	6.3	4.6
**2019 (excluded from the primary analysis)**	**1733**	**758 (43.7)**	**8.7**	**758**	4.7	2-week depressed mood	4.7	6.7	3.7	**100 (items not administered)**

Percentages of missing data are calculated within all women aged 40–64 years in that survey year. In 2019 the otolaryngological module was administered to a subsample, so the tinnitus item, self-rated hearing, and workplace-noise exposure were unavailable for 975 of 1733 women (56.3%), and the non-occupational noise items were not administered at all. Within the final analytic sample (2014–2018, N = 7239) the mental-health and sleep covariates were essentially complete: perceived stress 0 missing, depressive symptoms 7 (0.1%), physician-diagnosed depression 0, sleep duration 4 (0.1%), menopausal status 0, earphone use 7 (0.1%), non-occupational noise 3 (0.04%), impulse noise 108 (1.5%). The counts in the second column are all women aged 40 to 64 years responding in each survey year (8,170 in 2014–2018); they are therefore larger than the 7,903 shown in Figure 1, which additionally requires a valid integrated survey weight, primary sampling unit and stratum, and 267 women lacking one of these were excluded from every analysis. PHQ-9, Patient Health Questionnaire-9.

Table S2. Complete robustness analyses (Q4 versus Q1 adjusted prevalence ratios)

Analysis	n	aPR	95% CI	p
**Sequential adjustment**				
M0 Crude	7239	1.26	1.09–1.46	0.001
M1 + demographic, socioeconomic, behavioural, aerobic activity	7239	1.31	1.13–1.51	0.0003
M2 + auditory (self-rated hearing, workplace noise, occupation) — **primary**	7239	1.29	1.11–1.49	0.0007
M3 + cardiometabolic panel	6998	1.30	1.12–1.50	0.0004
M4 + mental health and sleep	7228	1.26	1.09–1.46	0.002
M5 + menopausal status	7239	1.29	1.12–1.49	0.0006
M6 + non-occupational noise	7122	1.29	1.12–1.49	0.0006
M7 + all covariate blocks simultaneously	7111	1.27	1.10–1.47	0.001
**Mental-health and sleep covariates entered individually (added to M2)**				
+ perceived stress	7239	1.26	1.09–1.46	0.002
+ depressive symptoms	7232	1.27	1.10–1.46	0.001
+ physician-diagnosed depression	7239	1.28	1.11–1.48	0.0008
+ sleep duration	7235	1.28	1.11–1.49	0.0008
+ all four together	7228	1.26	1.09–1.46	0.002
Per-quartile trend, mental-health-adjusted	7228	1.08	1.04–1.13	0.0004
**PHQ-9 years only (2014, 2016, 2018)**				
Primary model, PHQ-9 years	4355	1.37	1.14–1.65	0.001
Primary model + continuous PHQ-9 score	4355	1.32	1.09–1.59	0.004
**Outcome definition**				
Any current tinnitus (primary)	7239	1.29	1.11–1.49	0.0007
Bothersome tinnitus (annoying or sleep-disturbing)	6239	1.23	0.94–1.61	0.132
Sleep-disturbing tinnitus (72 cases)	5738	1.33	0.61–2.89	0.470
Excluding hearing-aid or cochlear-implant users	7224	1.29	1.11–1.49	0.0006
**Survey window and comorbidity**				
2014–2018 (primary)	7239	1.29	1.11–1.49	0.0007
2014–2019 (2019 included)	7974	1.25	1.08–1.44	0.002
Excluding diagnosed cardiovascular disease	7083	1.29	1.11–1.49	0.0007
**Auxiliary groups**				
Women aged 40–64 y (primary)	7239	1.29	1.11–1.49	0.0007
Men aged 40–64 y	5306	1.22	1.03–1.45	0.021
Women aged ≥65 y	3616	0.86	0.74–1.01	0.065

Sedentary-by-sex interaction p = 0.93; sedentary-by-age-group interaction p = 0.0008. aPR, adjusted prevalence ratio; CI, confidence interval; PHQ-9, Patient Health Questionnaire-9.

Table S3. Mental-health, sleep, and menopausal variables as correlates of current tinnitus in the primary model

Variable	aPR	95% CI	p
Perceived stress: a little (vs hardly any)	1.27	1.08–1.49	0.005
Perceived stress: much	1.78	1.48–2.13	<0.001
Perceived stress: very much	1.68	1.32–2.13	<0.001
Depressive symptoms (yes vs no)	1.33	1.16–1.53	<0.001
Physician-diagnosed depression (yes vs no)	1.43	1.22–1.67	<0.001
Sleep duration <6 h (vs 6–8 h)	1.12	0.99–1.28	0.075
Sleep duration >8 h (vs 6–8 h)	1.05	0.93–1.18	0.423
Postmenopausal (vs premenopausal)	0.86	0.72–1.01	0.071

Each variable was added separately to the primary model (age, education, household income, smoking, alcohol, aerobic activity, self-rated hearing, workplace noise, occupation). These estimates confirm that the added covariates are genuine correlates of tinnitus in this sample.

Table S4. Mean daily sedentary time by mental-health, sleep, and menopausal status

Variable	Level	Mean sedentary time, h/day (SE)
Perceived stress	Hardly any	7.07 (0.12)
	A little	7.10 (0.06)
	Much	7.42 (0.10)
	Very much	7.66 (0.23)
Depressive symptoms	No	7.15 (0.05)
	Yes	7.56 (0.17)
Physician-diagnosed depression	No	7.18 (0.05)
	Yes	7.26 (0.18)
Sleep duration	6–8 h	7.27 (0.06)
	<6 h	7.38 (0.13)
	>8 h	6.87 (0.09)
Menopausal status	Premenopausal	7.40 (0.08)
	Postmenopausal	6.98 (0.06)

Together with Table S3, these results show that the mental-health variables satisfy both arms of the confounder criterion (association with the exposure and with the outcome), which is why their minimal effect on the exposure estimate (Table S2) is informative rather than trivial.

Table S5. Sedentary time and tinnitus prevalence by occupational category

Occupational category	Mean sedentary time, h/day (SE)	Tinnitus prevalence, % (SE)
Managers and professionals	7.80 (0.12)	19.2 (1.6)
Clerical/office workers	9.19 (0.15)	20.3 (1.8)
Service and sales workers	6.28 (0.10)	17.0 (1.1)
Skilled agriculture/forestry/fishery	5.66 (0.20)	24.7 (2.9)
Craft and machine-operation workers	7.64 (0.24)	17.8 (2.5)
Elementary/manual labourers	6.10 (0.12)	23.9 (1.7)
Not economically active	7.43 (0.08)	25.2 (0.9)

Weighted estimates, Korean women aged 40–64 years, KNHANES 2014–2018 (N = 7239). Sedentary time varies substantially across occupational groups, but the ordering of occupations by sedentary time does not correspond to their ordering by tinnitus prevalence (for example, clerical workers have the longest sitting time and a below-average tinnitus prevalence, whereas agricultural workers have the shortest sitting time and an above-average prevalence). Occupation was adjusted for in all models.

Table S6. Distribution of tinnitus bothersomeness among women reporting current tinnitus

Bothersomeness	n	% of cases
Not bothersome	1000	63.6
Annoying / troubling	501	31.9
Difficult to sleep	72	4.6
Total cases	1573	100
